# A first look at the genome structure of hexaploid “Mitcham” peppermint (*Mentha × piperita* L.)

**DOI:** 10.1093/g3journal/jkae195

**Published:** 2024-11-19

**Authors:** Samuel C Talbot, Iovanna Pandelova, Bernd Markus Lange, Kelly J Vining

**Affiliations:** Department of Horticulture, Oregon State University, 4017 Agriculture and Life Sciences Building, Corvallis, OR 97331, USA; Department of Horticulture, Oregon State University, 4017 Agriculture and Life Sciences Building, Corvallis, OR 97331, USA; Institute of Biological Chemistry and M.J. Murdock Metabolomics Laboratory, Washington State University, Pullman, WA 99164-7411, USA; Department of Horticulture, Oregon State University, 4017 Agriculture and Life Sciences Building, Corvallis, OR 97331, USA

**Keywords:** Lamiaceae, *Mentha*, mint, peppermint, hexaploid, monoterpene

## Abstract

Peppermint, *Mentha × piperita* L., is a hexaploid (2*n* = 6*x* = 72) and the predominant cultivar of commercial mint oil production in the US. This cultivar is threatened because of high susceptibility to the fungal disease verticillium wilt, caused by *Verticillium dahliae*. This report details the first draft polyploid chromosome-level genome assembly for this mint species. The “Mitcham” genome resource will broaden comparative studies of disease resistance, essential oil biosynthesis, and hybridization events within the genus *Mentha*. It will also be a valuable contribution to the body of phylogenetic studies involving *Mentha* and other genera that contain species with varying ploidy levels.

## Introduction

Mints (*Mentha* spp.) are commercially grown for their distilled monoterpene-rich essential oils. Extracts are used for aroma and flavoring in a wide variety of consumer products ranging from cosmetics to candies, medicine, and dental care. The major species of import to industry are spearmints *Mentha spicata* (native spearmint) and *Mentha × gracilis* (Scotch spearmint) and peppermint (*Mentha* × *piperita*). The peppermint cultivar “Mitcham,” also called “Black Mitcham,” has been the most widely grown mint for much of the history of commercial production in the United States. First grown in England in the 1750s, then in colonial New England (Landing 1969), “Mitcham” is highly prized for its oil quality and yield. However, this clonally propagated herbaceous perennial is highly susceptible to the vascular wilt fungus *Verticillium dahliae*. “Mitcham” is also male sterile and not amenable to traditional breeding approaches, meaning introgression of resistance from other mints is not an option. Irradiation of “Mitcham” resulted in the release of 2 cultivars in the 1970s that showed higher field resistance to verticillium wilt, Todd's Mitcham and Murray Mitcham (Murray and Todd 1972; Todd *et al.* 1977), but these cultivars are not grown widely, and “Mitcham” continues to dominate the acreage for a crop with peppermint-like oil characteristics.

*M.* × *piperita* has a complex allohexaploid genome. It was first determined to be a natural hybrid of *Mentha aquatica* and *M. spicata* based on karyotype data (Harley and Brighton 1977), where chromosome numbers of *M.* × *piperita* were either (2*n* = 6*x* = 66) or, like “Mitcham,” (2*n* = 6*x* = 72). *Mentha spicata* itself has been shown to be a hybrid of *Mentha longifolia* and *Mentha suaveolens* by phylogenetic studies employing AFLP and chloroplast-based markers (Gobert *et al.* 2002, 2006). The AFLP results showed *M.* × *piperita* clustering closer to *M. aquatica* than to *M. spicata*, supporting the larger contribution of *M. aquatica* to the peppermint genome. The prevailing model of peppermint’s subgenome structure is one in which 4 sets of chromosomes were inherited from *M. aquatica* and 1 set each was inherited from *M. longifolia* and *M. suaveolens* via *M. spicata*. However, this subgenome structure remains to be definitively proven.

With advances in long-read DNA sequencing technology, plus concurrent advances in chromatin-based methods for chromosome scaffolding, larger, more complex plant genomes are being assembled to whole chromosome level, often including telomeres. This has been done for the accession of the diploid mint species used for genetic studies, *M. longifolia* CMEN 585 (PI 557767, local ID CMEN 535) (Vining *et al.* 2022). In that genome assembly, genes encoding key enzymes in the monoterpene biosynthesis pathway have been located on chromosomes. Still, polyploidy remains a challenge for chromosome scaffolding and haplotype phasing of plant genomes.

As a sterile allohexaploid, the genome of “Mitcham” is presumed to have not undergone meiotic recombination since it was first cultivated. Therefore, its subgenome structure is expected to be preserved. Here, we present the first draft genome assembly and gene annotation of “Mitcham” peppermint. It was produced through a combination of Pacific Biosciences HiFi sequencing and Omni-C-based scaffolding and contains partially resolved haplotypes.

## Materials and methods

### Plant material

Clonally propagated plant material from “Mitcham” peppermint (PI 557937, local ID CMEN 133) was obtained from the USDA National Clonal Germplasm Repository in Corvallis, OR, USA. It was maintained at the Oregon State University West Greenhouse complex by regularly transplanting a small wedge of soil containing stem and root material into fresh potting soil.

### Genome sequencing and assembly

High molecular weight genomic DNA was isolated from young, unexpanded leaf tissue using a modified cetyl trimethyl ammonium bromide method (Healey *et al.* 2014). Library preparation and sequencing on a Pacific Biosciences Sequel II instrument were done at Oregon CBD (Independence, OR). For high-throughput chromosome conformation capture (Hi-C), young, unexpanded leaf tissue was sent to Cantata Bio (Santa Cruz, CA, USA) for tissue processing, chromatin isolation, and Omni-C library preparation. Libraries were sequenced as 150 bp paired-end (PE) reads on an Illumina HiSeqX platform.

An initial genome size was estimated with a *k-mer* analysis of raw HiFi reads using jellyfish (version 2.3.0, RRID: SCR_005491) and the web version of GenomeScope (version 2.0, RRID: SCR_017014) with the following settings: *k-mer* length of 31, read length of 15,000 bp, and ploidy of 6. Raw PacBio HiFi reads were assembled using hifiasm (version 0.16.1-r375, RRID: SCR_021069) (Cheng *et al.* 2021). “Mitcham” was assembled using the –primary flag. The Arima Hi-C mapping pipeline (https://github.com/ArimaGenomics/mapping_pipeline) was followed to map Omni-C reads to the respective genome assembly. To scaffold the contig assemblies into pseudo-chromosomal scaffolds, YaHS (version 1.1, RRID: SCR_022965) was run using the Hi-C aligned, read name sorted bam file (Zhou *et al.* 2023). A Hi-C contact map was generated and manually curated by contig merging and rearrangement within JuiceBox (version 1.11.08, RRID: SCR_021172). This was done to obtain an accurate number of pseudomolecules for the species based on expected pseudomolecule size according to alignments of *M. longifolia* and suggested protocols by Lieberman-Aiden *et al.* (2009). Final assembly metrics were obtained using BUSCO (version 5.5.2, RRID: SCR_015008) in genome mode, using the Embryophyta_odb10 data set (Manni *et al.* 2021). To assess long-range structural variations, “Mitcham” was compared to the collapsed diploid assembly of *M. longifolia* V3 using minimap2 with divergence rate set to 20% (version 2.23-r1111, RRID: SCR_018550) and SyRI (version 1.6.3, RRID: SCR_023008) and visualized by plotsR (Goel *et al.* 2019; Li *et al.* 2021; Goel and Schneeberger 2022).

### Repeat identification

To create a repeat library of transposable element families, EDTA (version 2.1, RRID: SCR_022063) was used on the finalized genome assembly with the following parameters: –anno 1 –sensitive (Ou and Jiang 2019). EDTA is composed of a number of tools, including TIR-Learner, Generic Repeat Finder, HelitronScanner, and TEsorter. Low-complexity DNA sequences and repetitive regions identified by EDTA were soft masked prior to gene annotation. The quality of the genomes assembled repetitive regions was assessed using the long terminal repeat (LTR) assembly index (LAI); this pipeline was composed of LTRharvest in GenomeTools (version 1.6.1, RRID: SCR_016120), LTR_FINDER (version 1.2, RRID: SCR_015247), and LTR_retriever (version 2.9.6, RRID: SCR_017623) using suggested parameters to predict and combine likely full length candidate LTR-RTs (retrotransposons) (Xu and Wang 2007; Gremme *et al.* 2013; Xiong et al. 2014; Ou and Jiang 2018, 2019; Su *et al*. 2019; Shi and Liang 2019; Zhang *et al*. 2022). Calculation of the LAI was based on the following formula (Ou and Jiang 2018):


$$\mathrm{Raw}\;\mathrm{LAI}=\frac{\mathrm{Intact}\;\mathrm{LTR}\;\mathrm{retrotransposon}\;\mathrm{length}}{\mathrm{Total}\;\mathrm{LTR}\;\mathrm{sequence}\;\mathrm{length}}\;\times \;100$$

### Structural gene annotation

Gene prediction and annotation were facilitated by 150 bp PE Illumina RNA-Seq data derived from “Mitcham” root and stem tissue (PRJNA1144480) and concatenated with glandular trichome from *M.* × *piperita* (SRR1271557 and SRR1271558; Ahkami *et al.* 2015) and leaf tissue (SRR5150700; (Boachon *et al.* 2018) for a total of ∼380 million reads. Structural annotation of protein-coding genes was identified using the gene prediction software AUGUSTUS and GeneMark-ETP+, integrated by BRAKER3 (Gotoh 2008; Buchfink *et al.* 2015; Hoff *et al.* 2016; Hoff and Stanke 2019; Kovaka *et al.* 2019; Pertea and Pertea 2020). First, RNA-Seq reads were aligned using default parameters to the primary “Mitcham” chromosome-scaffolded assembly using the splice-aware aligner HISAT2 to generate a bam file (Kim *et al.* 2019). Second, BRAKER3 was run using the soft masked genome output from EDTA, the RNA-Seq bam file, and a curated Viridiplantae odb11 protein set (https://bioinf.uni-greifswald.de/bioinf/partitioned_odb11). For homology-based annotation, a protein set derived from the previous *M. longifolia* genome assembly (Vining *et al.* 2022) and 2 unpublished *M. longifolia* and *M. suaveolens* diploid assemblies consisted of 187,098 proteins and was used as input to BRAKER2 with GenomeThreader (Gremme *et al.* 2013) with AUGUSTUS ab initio and GALBA (Bruna et al. 2024); (Li 2023), separately. Gene predictions of the respective BRAKER3 and homology-based annotation runs were assessed for quality, deduplicated, and combined using TSEBRA with default settings (-c braker3.config) (Gabriel *et al.* 2021). Completeness of the predicted annotation sets was assessed using BUSCO protein mode with the Embryophyta odb10 data set. The proteome set was additionally vetted with OMArk (version 0.3.0) using default settings, the OMAmer Viridiplantae v2.0.0.h5 database, and a.splice file representing all alternatively spliced variants of transcripts (Nevers *et al.* 2024).

### Functional gene annotation

All gene models were subject to eggnog-v5 (version 5.0.2X; RRID: SCR_002456) functional annotation using default settings with DIAMOND and taxa limited to Viridiplantae (Hernández-Plaza *et al.* 2023). A single “best” haplotype was selected for improved functional analysis, consisting of 12 pseudo-chromosomal scaffolds with the highest quality alignment to *M. longifolia* V3. This set of chromosomes and their respective annotated gene models were subject to predictive functional analysis using within OmicsBox (version 3.1); the OmicsBox pipeline included CloudBLAST using BLASTx, InterPro, GO Merge, GO Mapping, and GO Annotation plus validation (Götz *et al.* 2008; Paysan-Lafosse *et al.* 2023). Conservation of putative high confidence gene models between assemblies was assessed using OrthoFinder (version 2.5.4, RRID: SCR_017118) (Emms and Kelly 2019).

## Results and discussion

### Genome assembly

A total of 1.9 million PacBio HiFi reads with an average length of 18,304 bp were generated from one 8 M SMRT cell, resulting in 34.8 Gb of sequencing data for *M. ×piperita* “Mitcham” (Supplementary Table 1). *k*-mer counts of the hexaploid “Mitcham” identified 1 distinct peak and a haplotype genome size estimate of 342 Mb (Supplementary Fig. 1). The first peak at 25 × is likely indicative of *k*-mers that are homozygous across subgenomes; the lack of other distinct peaks suggests insufficient read coverage (Ranallo-Benavidez *et al.* 2020). “Mitcham” was assembled using hifiasm and the –primary flag, generating 610 contigs for a total of 2,040 Mb and an N50 of 27.7 Mb (Table 1), representing 84% coverage of the 2.4 Gb estimated genome size based on GenomeScope results. Genome size estimates can vary by 10% or more in organisms with high repeat content; thus, the size is not outside expectation (Ranallo-Benavidez *et al.* 2020).

**Table 1.** Summary statistics for the *M. longifolia* and “Mitcham” peppermint genome assemblies.

Metrics	MlongV3	Mitcham
Size (Mb)	469	2,040
Number of contigs	326	610
Size (chr only, Mb)	462.6	1,974
N50 (Mb)	37.5	27.7
Longest length chr (Mb)	46.6	42
BUSCO (genome)	97.8%	94.2%*^a^*
LAI score	17.9	10.1
Number of protein-coding genes	42,107	247,493

^*a*^BUSCO score represents “best” 12 chromosomes assembled representative of a haplotype.

The hifiasm haplotype assemblies were used as inputs to the chromosome scaffolding process. The Omni-C protocol (Cantata Bio) was used to generate the sequencing library which was then scaffolded using YaHS (version 1.1) and manually curated within JuiceBox. Approximately 40 million PE 150 bp reads were generated for Hi-C, for a total yield of ∼12 Gb (6× coverage) (Supplementary Table 1). The average fragment insert size was 4,977 bp, indicating sufficient length for long-range scaffolding. The scaffolded chromosome-level assembly spans 1,974 Mb, capturing 96% of the hifiasm primary assembly. Following manual curation within JuiceBox based on minimap2 alignments to *M. longifolia* V3, 72 pseudo-chromosomal scaffolds were generated. BUSCO results in genome mode showed that the assembly was high quality and captured >98% of conserved genes in the embryophyta odb10 data set (Table 1).

### Characterization of repeats

To further vet the genome assembly, the LAI was calculated, wherein a LAI score less than 10 indicates draft quality, 10–20 as reference quality, and gold standard with an LAI score greater than 20 (Ou *et al.* 2018). Subsequent LAI analysis of the reference *M. longifolia* V3 was estimated at 17.9, whereas the LAI score of “Mitcham” was 10.13 and indicative of a draft genome assembly (Table 1).

Prior to gene annotation, genomic repeats were identified on all assemblies for downstream masking. The proportion of repeats and unknown elements identified by EDTA resulted in “Mitcham” having ∼62% of its genome masked (Supplementary Table 2). This proportion of repeats in “Mitcham” is significantly higher than *M. longifolia* V3 which had ∼45% of the genome masked. The majority of repeats were class I LTRs, with 32.3% of the genome composed of the Copia or Gypsy superfamily. A significant proportion of the genome (12.5%) consisted of helitron elements. Helitrons are unique transposable elements that are thought to significantly contribute to genome rearrangements, gene fragment duplications, exon shuffling, and chimeric transcript generation (Barro-Trastoy and Köhler 2024). Published helitron content estimates vary widely among plant species and between genotypes within a species; in the hexaploid wheat cultivar “AK58,” helitrons are made up 6% of total genome size, whereas in “Chinese Spring,” they are made up only 0.047% (Wang *et al.* 2022). Such a difference in helitron density could be attributed to computational difficulty in distinguishing helitron elements that are embedded in other repetitive sequences.

### Genome annotation

**Fig. 1.**
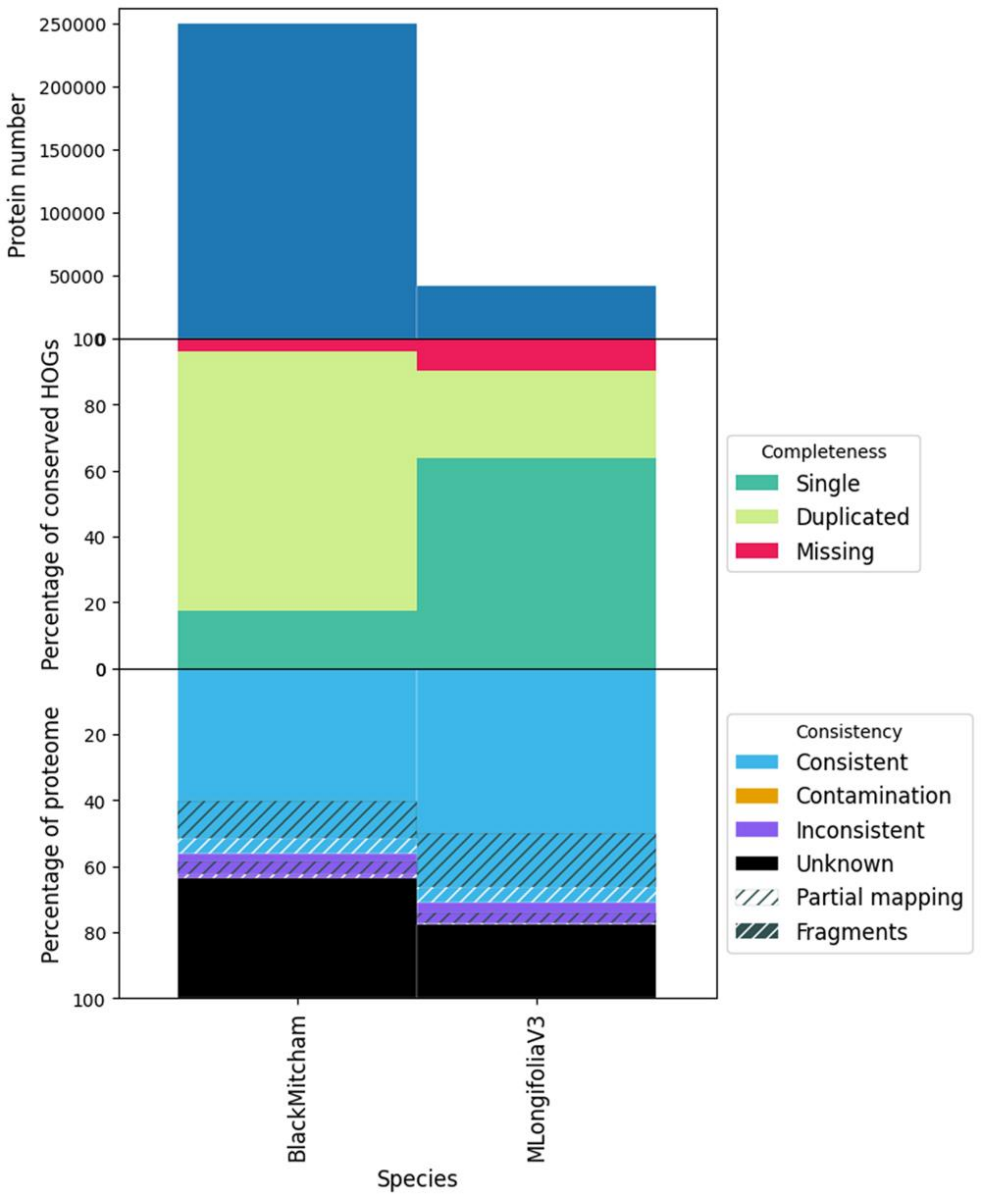
OMArk results of gene annotation sets of *M. longifolia* and “Mitcham.”

For “Mitcham,” 247,493 protein-coding genes were annotated in the genome assembly, representing roughly ∼41,200 genes per haplotype. The number of gene models in “Mitcham,” when considering an individual haplotype, is comparable to the unphased *M. longifolia* V3 assembly annotation of 42,107 genes. Translated coding sequences of putative gene models were subject to BUSCO analysis and revealed that the annotation set was 99.4% complete, although nearly all complete copies were duplications, which is expected due to polyploidy (Table 2). As an additional check, OMARK was used to compare *M. longifolia* V3 to “Mitcham”; the analysis showed that the majority of conserved hierarchical orthologous groups derived from the lamiids clade were duplicated in *M. × piperita*, with ∼36% of proteins having unknown lineage placement (Fig. 1). The full genome assembly was functionally annotated using eggnog, and 176,901 (71%) gene models were given a description. A set of 12 “Mitcham” scaffolds with the best alignment to the 12 *M. longifolia* V3 chromosomes was chosen to represent 1 “Mitcham” haplotype. This haplotype contained 43,758 gene models, which were then used as input to OmicsBox for a higher quality functional annotation. A total of 32,765 (∼75%) gene models were given a description by OmicsBox (Supplementary Fig. 2).

**Table 2.** Assessment of annotation completeness in *M. longifolia* and “Black Mitcham.”

Genome	Protein categories	BUSCO	
		Number	Percentage
*M. longifolia* (V3)	Complete BUSCOs (C)	1413	87.5
	Complete and single-copy BUSCOs (S)	1140	70.6
	Complete and duplicated BUSCOs (D)	273	16.9
	Fragmented BUSCOs (F)	95	5.9
	Missing BUSCOs (M)	106	6.6
	Total BUSCO groups searched	1614	100.0
Mitcham (primary)	Complete BUSCOs (C)	1605	99.4
	Complete and single-copy BUSCOs (S)	10	0.6
	Complete and duplicated BUSCOs (D)	1595	98.8
	Fragmented BUSCOs (F)	3	0.2
	Missing BUSCOs (M)	6	0.4
	Total BUSCO groups searched	1614	100.0

To assess gene models with shared homology, OrthoFinder was run with the annotated amino acid sequences derived from *M. longifolia* V3 and the “Mitcham” gene set. Approximately 77% (223,735) of Mitcham gene models were assigned orthogroups were categorized into orthogroups, with ∼63% of these being orthogroups with both species present (Supplementary Table 3). Of the species-specific orthogroups, the large majority (67%) of these orthogroups were composed of a single gene model. Nearly 66,000 (23%) of gene models were unassigned. The large number of single-copy orthogroups and unassigned gene models is indicative of both novel gene models and highly fragmented genes.

### Identification of subgenomes within “Mitcham”

**Fig. 2.**
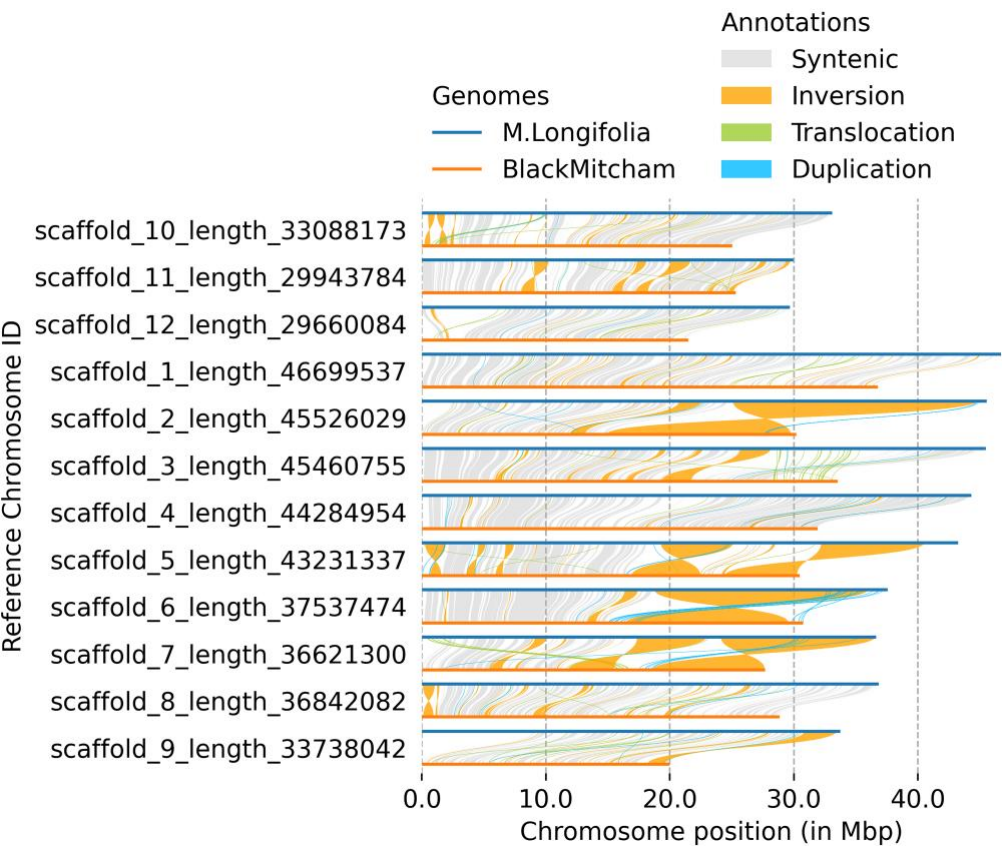
SyRI plot of 12 “Mitcham” pseudo-chromosomes against the *M. longifolia* V3 genome assembly.

The ultimate goal of this research project is to identify the distinct subgenomes contributed to “Mitcham” peppermint by the 3 ancestral species. The single distinct peak observed in the GenomeScope result is indicative of insufficient sequencing depth to resolve haplotypes. In an initial step, the 12 highest quality “Mitcham” pseudo-chromosome scaffolds were identified based on alignment to those of *M. longifolia* V3 using minimap2 and SyRI; then, pairwise alignments were plotted using plotsr. Only ∼40% of the total length of the “Mitcham” scaffolds aligned meaningfully to those of *M. longifolia* (Supplementary Tables 4 and 5). Of the syntenic regions between the 2 genomes, the majority of alignments contained inversions (Fig. 2). The low synteny was expected for several reasons. First, the “Mitcham” subgenome haplotypes are only partially resolved, and no additional sequence data from other ancestral species were used to define haplotypes. Second, the specific representatives of the hypothesized ancestral species that hybridized in nature are unknown, and these species are underrepresented in germplasm collections. Third, the *M. longifolia* diploid “CMEN 585” is from South Africa and is assumed to not be a direct ancestor of “Mitcham,” which reportedly arose in Europe. Given the limited genome resources for *Mentha*, this exploratory study was not intended to provide a clear definition of subgenomes, but rather to highlight the need to generate genome sequence data from sets of accessions representing the range of diversity within each species.

The results reported here open the door for further exploration of the origin of “Mitcham” peppermint, including definition of subgenomes by species of origin, as has been done for the allo-octoploid cultivated strawberry, *Fragaria × ananassa* (Edger *et al.* 2019). However, unlike the sexually fertile cultivated strawberry, the chromosomes in the subgenomes of “Mitcham” are presumed to be frozen in time, awaiting discovery.

## Supplementary Material

jkae195_Supplementary_Data

## Data Availability

The genome assembly can be found at under NCBI BioProject PRJNA1090951. The related annotation files are available as downloadable tracks through a publicly accessible genome browser: https://mintgenomes.oregonstate.edu. These files with additional scripts that were used to assemble the genome are also accessible through our GitHub at https://github.com/ViningLab/ViningLab_code/blob/3289b81866d0e01c4fa824e460c42e2f0c5b126e/G3_BM_AssemblyPipeline. Supplemental material available at G3 online.
