# Supplementary material for: A first look at the genome structure of hexaploid “Mitcham” peppermint (Mentha × piperita L.)
Source: G3 (Bethesda). 2024 Nov 19;14(12):jkae195. doi: 10.1093/g3journal/jkae195 (PMC11631389; doi:10.1093/g3journal/jkae195)

Supplement File.

Table S1. Summary of *Mentha spp.* sequencing data from PacBio and Omni-C platforms.

Table S2. *'Black Mitcham'* 'V1' primary assembly EDTA output.

Table S3. OrthoFinder results comparing 'Black Mitcham' and *M. longifolia* V3 gene model sets.

Table S4. SyRI Structural Annotations of 'Black Mitcham' as reference and *M. longifolia* V3 as query.

Table S5. SyRI Sequence Annotations of 'Black Mitcham' as reference and *M. longifolia* V3 as query.

Figure S1. GenomeScope results of raw PacBio HiFi *M. xpiperita* 'Black Mitcham' reads.

Figure S2. OmicsBox summary metrics for functional annotation of one *M. xpiperita* 'Black Mitcham' haplotype.

Table S1. Summary of 'Black Mitcham' sequencing data from PacBio and Omni-C platforms.

Sequencing platform	Insert length	Sequencing model	Number of reads	Total nucleotides
PacBio Sequel IIe	>20kb	1x 8M SMRT cell	1.93 M	34.8 Gb
Omni-C on HiSeqX	300bp	2x150	40.4 M	12.1 Gb

Table S2. *'Black Mitcham'* 'V1' primary assembly EDTA[1] output.

Class	Number of elements	Length (bp)	Percentage of genome
**LTR**	**679,389**	**659,871,672**	**32.33%**
Copia	204,196	260,067,812	12.74%
Gypsy	267,457	283,508,142	13.89%
unknown	207,736	116,295,718	5.70%
**TIR**	**956,227**	**339,698,800**	**16.65%**
CACTA	63,568	21,158,517	1.04%
Mutator	192,359	53,173,408	2.61%
PIF_Harbinger	44,350	19,357,142	0.95%
Tc1_Mariner	510,361	194,511,627	9.53%
hAT	145,589	51,498,106	2.52%
**nonLTR**	**1,920**	**1,529,864**	**0.07%**
LINE_element	1,067	666,513	0.03%
unknown	853	863,351	0.04%
**nonTIR**	--	--	--
helitron	**754,485**	**255,121,566**	**12.50%**
**repeat_region**	**87,955**	**24,182,197**	**1.19%**
**Total Genome Masked**	**2,479,976**	**1,280,404,099**	**62.75%**

[1]EDTA run with parameters: --sensitive 1 --analysis 1.

Table S3. OrthoFinder results comparing 'Black Mitcham' and *M. longifolia* V3 gene model sets.

OrthoFinder Category	Count
Number of genes	289,578
Genes in Orthogroups	223,735
Number of unassigned genes	65,853
Number of Orthogroups	39,603
Number of species-specific Orthogroups	14,4661
Number of genes in species-specific orthogroups	120,179
Number of Orthogroups with all species present	24,942
Number of single-copy orthogroups	9,873

Table S4. SyRI comparisons of chromosome structure derived from alignments using a 'Black Mitcham' haplotype as reference and *M. longifolia* V3 as query.

Structural Annotations	Count	Reference length (bp)	Query length (bp)
Syntenic regions	1,023	158,176,085	138,995,928
Inversion	214	94,876,643	74,841,876
Translocations	1,214	5,391,770	5,412,095
Duplications (reference)	519	2,839,865	N/A
Duplications (query)	671	N/A	2,842,068
Not aligned (reference)	2,915	201,509,951	N/A

Table S5. SyRI comparisons of sequence annotations derived from alignments using a 'Black Mitcham' haplotype as reference and *M. longifolia* V3 as query.

Sequence Annotations	Count	Reference length (bp)	Query length (bp)
SNPs	892,386	892,386	892,386
Insertions	89,362	N/A	980,070
Deletions	79,130	1,005,361	N/A
Copygains	156	N/A	222,220
Copylossers	131	466,248	N/A
Highly diverged	5,313	218,682,349	179,754,399
Tandem repeats	1	299	549

Figure S1. GenomeScope results of raw PacBio HiFi *M. xpiperita* 'Black Mitcham' reads.

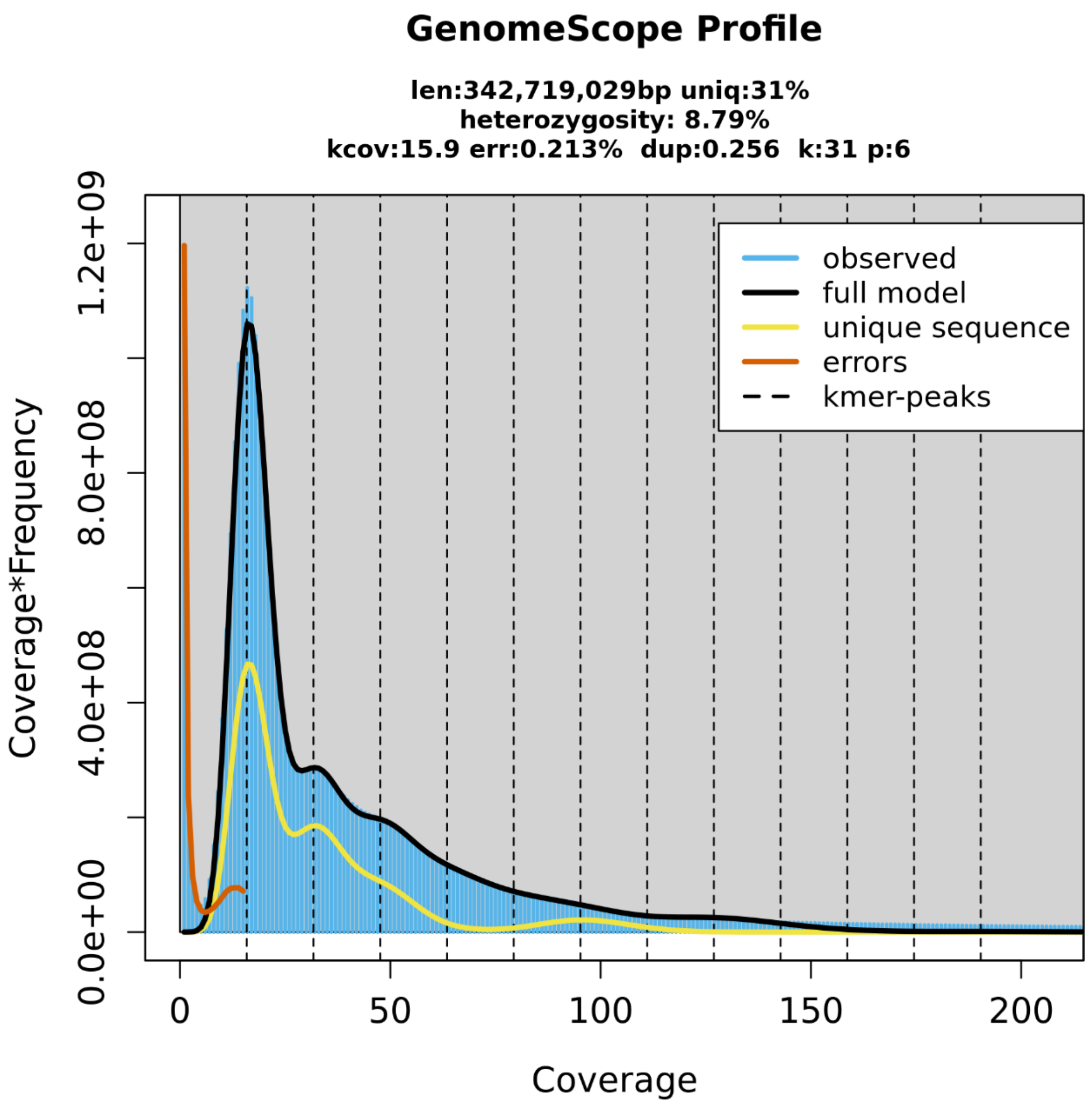
GenomeScope Profile
len:342,719,029bp uniq:31%
heterozygosity: 8.79%
kcov:15.9 err:0.213% dup:0.256 k:31 p:6
observed
full model
unique sequence
errors
kmer-peaks
Coverage*Frequency
0.0e+00
4.0e+08
8.0e+08
1.2e+09
0
50
100
150
200
Coverage

Figure S2. OmicsBox summary metrics for functional annotation of one *M. xpiperita* 'Black Mitcham' haplotype. Pie chart shows total distribution of OmicsBox functional annotation performed on amino acid gene models.

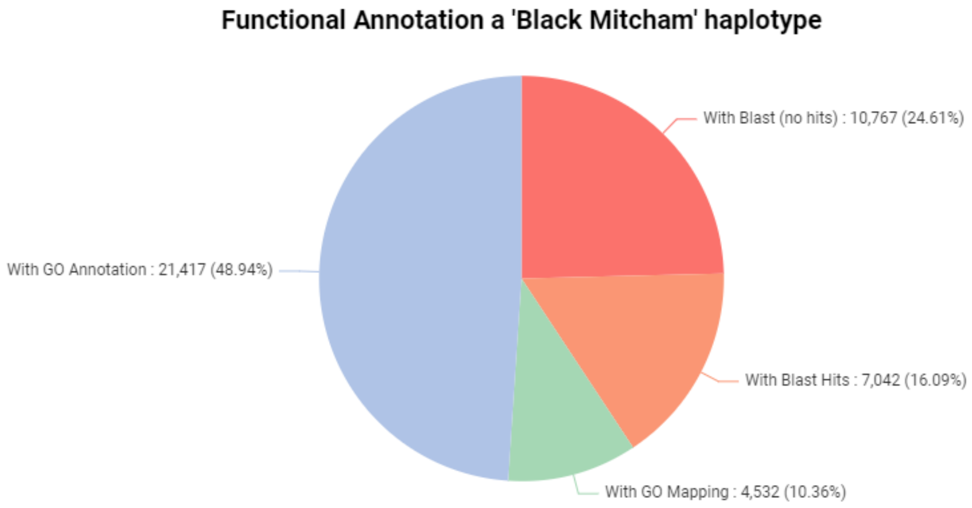

Supplement: jkae195_Supplementary_Data [file jkae195_supplementary_data.pdf]
